# Extensive paternal mtDNA leakage in natural populations of *Drosophila melanogaster*

**DOI:** 10.1111/mec.12256

**Published:** 2013-03-04

**Authors:** Maria D S Nunes, Marlies Dolezal, Christian Schlötterer

**Affiliations:** Institut für PopulationsgenetikVetmeduni Vienna, Veterinärplatz 1, A-1210, Vienna, Austria

**Keywords:** *Drosophila melanogaster*, heteroplasmy, mtDNA diversity, paternal leakage, recombination

## Abstract

Strict maternal inheritance is considered a hallmark of animal mtDNA. Although recent reports suggest that paternal leakage occurs in a broad range of species, it is still considered an exceptionally rare event. To evaluate the impact of paternal leakage on the evolution of mtDNA, it is essential to reliably estimate the frequency of paternal leakage in natural populations. Using allele-specific real-time quantitative PCR (RT-qPCR), we show that heteroplasmy is common in natural populations with at least 14% of the individuals carrying multiple mitochondrial haplotypes. However, the average frequency of the minor mtDNA haplotype is low (0.8%), which suggests that this pervasive heteroplasmy has not been noticed before due to a lack of power in sequencing surveys. Based on the distribution of mtDNA haplotypes in the offspring of heteroplasmic mothers, we found no evidence for strong selection against one of the haplotypes. We estimated that the rate of paternal leakage is 6% and that at least 100 generations are required for complete sorting of mtDNA haplotypes. Despite the high proportion of heteroplasmic individuals in natural populations, we found no evidence for recombination between mtDNA molecules, suggesting that either recombination is rare or recombinant haplotypes are counter-selected. Our results indicate that evolutionary studies using mtDNA as a marker might be biased by paternal leakage in this species.

## Introduction

The mechanism of mitochondrial inheritance is far from being fully understood, but some evidence suggests that the transmission of animal paternal mtDNA is prevented by stochastic loss due to lower mtDNA content in sperm than in oocytes (Hecht *et al*. 1984; Piko & Taylor 1987; Cree *et al*. 2008; Wolff & Gemmell 2008) and/or by recognition and elimination mechanisms elicited by the egg (Hiraoka & Hirao 1988; Kaneda *et al*. 1995; Sutovsky *et al*. 1999, 2003; Thompson *et al*. 2003; Al Rawi *et al*. 2011; Sato & Sato 2011; DeLuca & O'Farrell 2012). Early reports of paternal leakage came from studies of hybridization between species or subspecies where these mechanisms might be relaxed (Lansman *et al*. 1983; Kondo *et al*. 1990; Gyllensten *et al*. 1991; Meusel & Moritz 1993; Kaneda *et al*. 1995; Shitara *et al*. 1998; Kvist *et al*. 2003). More recently, however, cases of intraspecific paternal leakage were also detected in humans (Schwartz & Vissing 2002), scorpions (Gantenbein *et al*. 2005), flies (Kondo *et al*. 1992; Sherengul *et al*. 2006; Wolff *et al*. 2013) and lizards (Ujvari *et al*. 2007).

In *Drosophila,* paternal leakage of mtDNA was first detected in a *Drosophila simulans* population from Réunion (Satta *et al*. 1988; Matsuura *et al*. 1991), where it could have resulted from interspecific hybridization between *D. simulans* and *Drosophila mauritiana*. Later, another study found that African *D. simulans* populations polymorphic for siII and siIII mtDNA [two highly divergent haplotypic classes occurring in this species (Ballard 2000)] have up to 6% of individuals carrying both haplotypes (Dean *et al*. 2003). As hybridization with *D. mauritiana* is unlikely in this geographical region, these heteroplasmic individuals were assumed to be the outcome of intraspecific paternal leakage. Recently, Wolff *et al*. (2013) observed paternal leakage at a frequency of 0.66% in experimental crosses using *D. simulans* strains collected from East African populations with documented heteroplasmy. In *Drosophila melanogaster*, however, no heteroplasmy has been documented in natural populations.

While intraspecific paternal leakage of mtDNA could be due to rare events of recognition failure, it is also possible that transmission of small amounts of paternal mtDNA is common but our ability to detect it is limited by insufficient mtDNA differentiation, as well as low sensitivity of the detection methods.

Estimates of the frequency of paternal mtDNA leakage in natural populations are essential to understand its impact on the evolution of mtDNA. In particular, the fact that animal mitochondria are able to fuse, generating a connective network (Cortese 1999; Yaffe 1999), and have the necessary toolkit of enzymes to recombine (Thyagarajan *et al*. 1996; Lakshmipathy & Campbell 1999a,b) opens the possibility for heterologous recombination to occur. This could allow for new combinations of favourable alleles to arise as well as deleterious mutations to be purged, a mechanism that would be in clear disagreement with the model of uniparental inheritance. In turn, this could also have a significant impact on phylogenetic and demographic inference based on mtDNA, because its use as a molecular marker relies heavily on its strict maternal transmission and absence of recombination (see e.g. White *et al*. 2008 for a recent review). Despite the mounting number of studies reporting paternal leakage of mtDNA, very few have attempted to estimate its frequency in natural populations (e.g. Pearl *et al*. 2009).

The identification and quantification of paternal leakage in natural populations is highly dependent on haplotype diversity. Only if paternal and maternal mtDNA differs in sequence, heteroplasmy caused by paternal leakage can be detected. Previous work revealed high mitochondrial diversity in *D. melanogaster* populations of the European/Mediterranean region (Nunes *et al*. 2008a), which makes them a good target to investigate the occurrence of paternal leakage in this species. In this study, we use real-time quantitative PCR (RT-qPCR) to determine the frequency of heteroplasmy in these natural populations and study the transmission of heteroplasmy from mother to offspring.

## Materials and methods

### Sequence data

Previously, we surveyed variability in natural *Drosophila melanogaster* populations for a 554-bp fragment of the COI gene (Nunes *et al*. 2008a,b). Here, we expanded the sample size for many of the European/Mediterranean populations. In total, we obtained sequence data for 263 individuals from 26 populations. For comparison, we added 10 individuals from three North American populations, which were also sequenced for COI (Table S1, Supporting information). All flies used in this study originated from isofemale lines established from wild-caught females. Most samples were only available as ethanol-preserved specimens.

Genomic DNA of a single fly was isolated for each line, using the high salt extraction method (Miller *et al*. 1988). Amplification was carried out with the primers listed in Table S2 (Supporting information) using standard amplification conditions and primer annealing temperatures as provided in Table S2 (Supporting information). PCR products were purified using 96-well plates (Millipore, Billerica, MA, USA) according to the supplier's protocol. All PCR products were sequenced in both directions with the primers used for the fragment amplification, using ET Dye Terminator Sequencing Chemistry (GE Healthcare Bio-Sciences, Piscataway, NJ, USA). Nonincorporated dyes were removed using Sephadex G-50 fine (GE Healthcare Bio-Sciences), and the sequencing reaction products were separated on a MegaBACE 500 automated capillary sequencer (GE Healthcare Bio-Sciences). Sequences were edited and assembled using AutoAssembler3.1 and CodonCode Aligner3.0.3.

To test recombination, a subset of 55 individuals from the European/Mediterranean populations was sequenced for six additional fragments of the mtDNA molecule (primer sequences and annealing temperature, as well as PCR product sizes, are given in Table S2, Supporting information). To maximize the chance of detecting recombinants, we included 34 additional individuals from other geographic regions, as well as old stock centre flies (Table S1, Supporting information), because some of the haplotypes restricted to Europe/Mediterranean were likely to be globally distributed until recently (Nunes *et al*. 2008b). We found haplotype inconsistency between different mtDNA fragments (ND2 and COI, for example) in a few individuals (ROME3, GOT11 and SZ13). While this could be indicative of recombination, the fact that one of the fragments (ATPase6) showed heteroplasmy (for ROME3 and GOT11) suggested that these individuals are in fact heteroplasmic. To prove that haplotype inconsistencies between loci are an artefact of preferential PCR amplification of one allele, we designed new primers. Our new analyses confirmed that polymorphism in the primer binding site decreased the efficiency of amplification of one of the mtDNA variants. In addition, we found two heteroplasmic sites in the sequences of TU19. As the sequences of four individuals (ROME3, GOT11, SZ13 and TU19) could give a spurious signal of recombination, all heteroplasmic individuals were removed from further analysis.

### Sequence data analysis

Standard diversity estimates were calculated using DnaSP version 5.10 (Librado & Rozas 2009) for each European/Mediterranean population using the COI data.

To investigate the possibility of nuclear insertions of mtDNA (NUMTs) resulting in spurious inference of recombination, we searched for nuclear copies of the gene fragments analysed in our survey using blastn (Altschul *et al*. 1997) against the *D. melanogaster* genome (release 5.25). The largest hit (146 bp) to the genome was on the 4th chromosome with an e-value of 1 × e^−28^, and it showed no sequence conservation to the PCR primers used in our survey.

Recombination was tested on a concatenated haplotype data set (a total of 3.7 kb) obtained from the sequencing of seven mtDNA fragments in 85 individuals (see above). In addition to the 37 haplotypes identified in these 85 individuals, we added to the data set the sequences of the two mitochondrial genomes available from GenBank (Table S1, Supporting information). The minimum number of recombination events and the number of pairs of sites with four gametic types were estimated following Hudson & Kaplan (1985) as implemented in DnaSP version 5.10 (Librado & Rozas 2009). We also calculated the Pairwise Homoplasy Index (PHI, Bruen *et al*. 2006) using SplitsTree v4.11 (Huson & Bryant 2006). This test estimates the probability that recombination has occurred by looking at the genealogical correlation between pairs of sites taking physical distance into account. It can therefore be used to distinguish recombination from recurrent mutation as homoplasies due to recurrent mutation, in contrast to those resulting from recombination, should not correlate with distance. The PHI test was previously shown to be powerful to detect recombination provided that the data set has sufficient variation (White *et al*. 2008). In addition, this test is robust to the presence of mutation hotspots as well as to violations of common assumptions, such as random mating and constant population size (Bruen *et al*. 2006).

Minimum spanning networks for the COI and the recombination data sets were constructed using the software tcs (Clement *et al*. 2000), which implements the method of probability of parsimony (Templeton *et al*. 1992).

### Quantification of heteroplasmy

We used allele-specific RT-qPCR with a TaqMan® probe (Steinborn *et al*. 1998) to estimate heteroplasmy in individual flies. DNA was extracted from single flies as described above. Heteroplasmy was determined as the proportion of the lower-frequency mtDNA haplotype L (HapL) present in an individual. We designed universal primers (1812F, 1901R) to amplify all mtDNA haplotypes and allele-specific primers (1756AF and 1756TF) that differ in position 1779 to allow discrimination between two groups of COI haplotypes. Both primers have an additional mismatch in position 1777 to increase specificity (Newton *et al*. 1989; Wu *et al*. 1989). The same reverse primer (1901R) was used for the three different assays. Hot-start amplification reactions containing 4 mm MgCl_2_, 0.2 mm dNTPs, 300 nm of each primer, 1× Taq polymerase buffer, 1 unit of hot-start Taq polymerase, 100 nm TaqMan probe (FAM/BHQ1 labelled) and 10–50 ng total cellular DNA were performed in a ABI PRISM 7900 sequence detection system (PE Applied Biosystems, Germany). Primers and probe sequences can be found in Table S3 (Supporting information). Numbering refers to GenBank entry AF200828. Each run consisted of total (1812F and 1901R) and haplotype group–specific (1756AF or 1756TF and 1901R) assays and was done in duplicates for samples, standard curve (described below) and negative controls. The following amplification conditions were used: initial denaturation of 15 min at 95 °C followed by 45 cycles of denaturation at 95 °C for 20 s, primer annealing at 45 °C for 30 s and primer extension at 60 °C for 30 s. The threshold cycle (*C*_t_) values were determined using the Sequence Detector 2.3 software (PE Applied Biosystems). The quantity *X* of the less frequent mtDNA haplotype L in an individual *i* was normalized by a reference (homoplasmic individual with haplotype L) as determined by the equation:


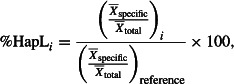


where 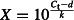 and 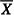 is the average *X* between duplicates, *specific* refers to haplotype-specific quantification and *total* to the total mtDNA quantification, *k* is the slope and *d* is the intercept of a standard curve generated by an eightfold serial dilution given by *C*_t_ = *k* × log (dilution factor) + *d*. For each run, the dilution series was done with the corresponding reference sample. Samples were considered positive (i.e. heteroplasmic) when %HapL ≥ 0.3 and *C*_t_ values were within the range of the standard curves. Standard deviation (SD) values (of *C*_t_ values) between duplicates were low (Table S4, Supporting information).

### Heteroplasmy screen in natural populations

Using RT-qPCR, the frequency of heteroplasmy in natural populations was estimated based on a screen of 66 randomly chosen individuals from 25 European/Mediterranean populations (Table S4, Supporting information).

### Rate of paternal leakage and distribution of heteroplasmy in the offspring of a heteroplasmic female

We performed 38 experimental crosses between individuals of the EVO population (where some of the lines have heteroplasmic individuals) carrying different mtDNA haplotypes distinguishable by our allele-specific RT-qPCR assay (see Table S5, Supporting information, for a detailed overview). These crosses allowed us to (i) investigate the rate of paternal leakage and (ii) study the transmission of heteroplasmy from a heteroplasmic mother to its offspring. This was achieved in the following way: four individuals from the progeny of each cross were assayed by RT-qPCR. Given the large number of offspring analysed, we performed an initial screen without standard curves to identify potential heteroplasmic offspring. This was assessed based only on the difference in *C*_t_ (Δ*C*_t_) between the total and the allele-specific assay. An individual was judged as putatively heteroplasmic if its Δ*C*_t_ was smaller or equal to 14 (this empirical threshold was based on the heteroplasmy screen in natural populations). When putatively heteroplasmic progeny was detected, the mother was also assayed. In all cases where heteroplasmic offspring was found (nine crosses), the mother was also heteroplasmic.

To estimate the variation in haplotype frequencies among the offspring of heteroplasmic females, we analysed ∼20 F1 offspring in three of the nine crosses with heteroplasmic mothers. Crosses C2RR2, C4RR2 and C9RR3 were chosen because they had the largest number of heteroplasmic offspring and with the highest heteroplasmy levels. Individual male offspring and mothers were tested with RT-qPCR, as described in the section Quantification of heteroplasmy, to estimate %HapL.

In addition to offspring from laboratory crosses, we also determined the distribution of haplotypes in offspring from a female that was inseminated in the wild. We analysed the offspring of three heteroplasmic females (based on COI sequences): KR6, MOSKAU104 and NEU1 (sample size for each cross can be found in Table S6, Supporting information).

Using an analysis of median scores (*proc NPAR1WAY* option *median* implemented in sas 9.2; SAS Institute INC. Cary, NC, USA; 2008), we tested for location differences in the heteroplasmy distributions between groups. This nonparametric approach was chosen because residuals were not normally distributed and showed variance heterogeneity. First, we tested for differences in the distribution of heteroplasmy between sexes within and across KR6, MOSKAU104 and NEU1 progeny. We also contrasted ‘natural populations’ (KR6, MOSKAU104 and NEU1) against ‘crosses’ (C2RR2, C4RR2 and C9RR3), as well as all six lines against each other within and across sexes. Finally, the level of heteroplasmy in the progeny of the three crosses was compared with that of the corresponding mother. We did not perform any correction for multiple testing because no significant differences between groups were detected. The data file is available in Table S6 (Supporting information).

If the transmission of heteroplasmy is a random process, we can use a modification of Wright's genetic drift model (Wright 1968) to estimate the time, in number of generations (*n*), required for all the descendants of a heteroplasmic female to return to the homoplasmic state (nloss). This modified model was first applied to the study of heteroplasmy transmission in *Drosophila mauritiana* by Solignac *et al*. (1984). Here, we follow Rand & Harrison (1986), who have applied the model to single generation data:


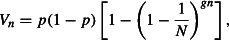


where *V*_*n*_ is the variance at the *n*th generation of the distribution of heteroplasmy in the offspring of the six lines studied (KR6, MOSKAU104, NEU1, C2RR2, C4RR2 and C9RR3); *p* is the initial frequency of heteroplasmy, that is, the maternal heteroplasmy level (for KR6, MOSKAU104 and NEU1; we used the mean heteroplasmy frequency of the F1 individuals because the mothers were not available); *N* is the estimated number of segregating mitochondria, and *g* is the number of germ-cell generations per animal generation (following Solignac *et al*. (1984), we used *g* = 10). Heteroplasmy is lost when 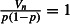. As mentioned by Rand & Harrison (1986), if *V*_*n*_ is calculated to many decimal places (we use six decimal places), nloss can become arbitrarily large. Therefore, we also calculated nhalf, which is the number of generations necessary to decrease by half the difference between the initial and the final variance.

We used Hedrick's forward and backward mutation equation (eq. 8.1 in Hedrick 2000) to estimate the rate of paternal leakage. If the equilibrium frequency (*q*_*e*_) is the observed frequency of heteroplasmy in natural populations and the rate of backward mutation (*v*) is the rate of heteroplasmy loss estimated from the distribution of heteroplasmy in offspring of a heteroplasmic mother, then the rate of mutation (*u*) will be the rate of paternal leakage occurring in natural populations.

## Results

**Fig. 1:**
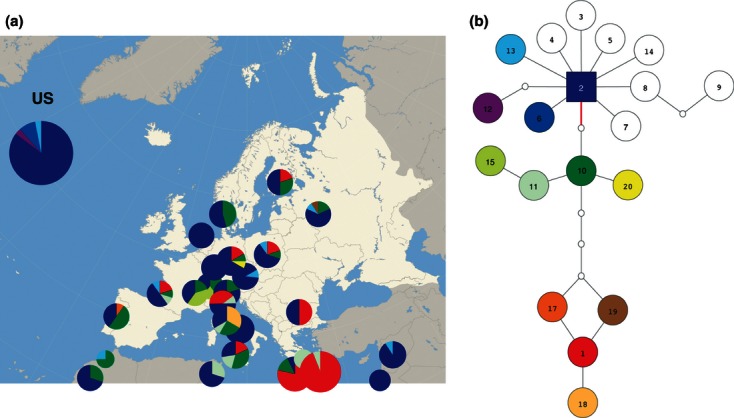
COI haplotypic diversity in European/Mediterranean populations of *Drosophila melanogaster*. (A) The distribution of haplotypes per population. Pie size is proportional to sample size. Colours are used to distinguish haplotypes according to (B). Haplotypic diversity in three North American populations (US label in the Figure) is shown for comparison. (B) The haplotype network of COI haplotypes (identified by numbers inside the circles and filling colours). Noncoloured circles correspond to haplotypes identified previously (Nunes *et al*. 2008a) but not detected in the samples added in this study. Small circles in the branches refer to the number of mutation steps. The SNP (T/A) at position 1779 assayed in the RT-qPCR occurs in the branch highlighted in red; haplotypes above and below the branch contain a T and an A, respectively.

Previously, we showed that European/Mediterranean *Drosophila melanogaster* populations harbour more mtDNA variation than populations from other regions of the world, including Africa (Nunes *et al*. 2008a). This difference in mtDNA variability does not reflect the demographic past, but is the outcome of *Wolbachia* infection dynamics (Nunes *et al*. 2008b). To increase the probability to detect heteroplasmy, we increased the sample size for European/Mediterranean populations, compared with previous studies. Twenty-one populations had more than a single COI haplotype (Fig. 1), with haplotype diversities ranging from 0.13 to 0.80 (Table S7, Supporting information). Careful analysis of the sequence chromatograms indicated that four samples, MOSKAU104, KR6, KR15 and NEU1, showed obvious signs of heteroplasmy, suggesting that natural *D. melanogaster* populations have heteroplasmic individuals at a detectable frequency.

### Origin of heteroplasmy

Heteroplasmy of mtDNA could arise from three different mechanisms: (i) new mutations that are segregating within a cell/individual, (ii) NUMTs and (iii) paternal leakage. Interestingly, while the previously reported mtDNA heteroplasmy in *D. melanogaster* (Hale & Singh 1986; Townsend & Rand 2004) was probably caused by intramolecular slippage like mutations of the repeats in the AT-rich control region, in our study heteroplasmy was not restricted to a single site, but could be detected at multiple positions. This suggests that heteroplasmy was not generated by mutation, but through paternal leakage. Indeed, heteroplasmy could in all cases (MOSKAU104, KR6, KR15 and NEU1) be explained by the combination of two diverged haplotypes (e.g. Fig. S1, Supporting information). It is also unlikely that NUMTs explain the observed heteroplasmy because only three NUMTs of a total length of 724 bp have been reported in the *D. melanogaster* genome (Bensasson *et al*. 2001), and we also could not find any significant hit in the reference genome (release 5.25) when searching for nuclear copies of the gene fragments analysed in our survey. In addition, the heteroplasmic sites found in our samples spanned, in some cases, the whole COI fragment sequenced. Altogether, these results indicate that paternal leakage is the cause of the observed SNP heteroplasmy in *D. melanogaster*.

### Natural populations of *Drosophila melanogaster* have a high frequency (14%) of heteroplasmic individuals

The detection of heteroplasmy by DNA sequencing is challenging if one of the haplotypes occurs at low frequency. Hence, we developed an allele-specific real-time quantitative PCR (RT-qPCR) assay, which also detects low-frequency variants. We selected a SNP (T/A) at position 1779 (relative to AF200828, Fig. 1B) for the assay, as both alleles occur at a similar frequency in European populations, thus maximizing the power to detect heteroplasmy.

We analysed 66 randomly selected individuals from 25 populations and found nine individuals (14%) with a second mtDNA haplotype. The minor allele is T in seven of these nine individuals and A in the remaining two individuals. The average frequency of the minor allele is 0.8% (see Table S5, Supporting information for a comprehensive overview of the results). Despite the overall low levels of the second allele, it was possible in some cases to confirm the heteroplasmy by direct sequencing of other fragments of the mtDNA molecule (e.g. ATPase6 for AG21, data not shown).

### No evidence for recombination in *Drosophila melanogaster* mtDNA

To determine whether the high rate of heteroplasmy could result in recombination among the different mtDNA haplotypes, we sequenced six more mtDNA fragments in several samples (Table S1, Supporting information). We identified four additional heteroplasmic individuals (GOT11, ROME3, SZ13 and TU19, see Materials and methods), which were excluded from the analysis. The mtDNA of the remaining 85 individuals can be grouped into 37 different haplotypes (Fig. S2 and Table S1, Supporting information).

Given the high frequency of heteroplasmy, it is possible that recombination between different mtDNA haplotypes occurs and can be detected in nonheteroplasmic individuals. In a first attempt to test for recombination, we used Hudson's four-gamete test (Hudson & Kaplan 1985) and detected a minimum of eight recombination events. Nevertheless, this test cannot distinguish homoplasies created by recurrent mutations from those created by recombination events. As recurrent mutations should only affect a single nucleotide, while recombination results in the exchange of multiple sites, we inspected putative recombination events and found all of them to be compatible with recurrent mutation affecting only a single site. For a formal test, we applied the Pairwise Homoplasy Index test (PHI, Bruen *et al*. 2006) but again failed to find support for recombination, confirming the results of our visual inspection.

### Experimental detection of paternal leakage and study of heteroplasmy transmission from mother to offspring

The high proportion of heteroplasmic individuals in our study could either result from rare paternal leakage combined with a highly stable transmission or from frequent paternal leakage with high rates of drift.

If the rate of paternal leakage is very high, we might be able to reproduce it experimentally. We performed 38 crosses between individuals of the EVO population carrying haplotypes distinguishable by our allele-specific RT-qPCR assay (Table S5, Supporting information) and found no evidence for new paternal leakage. This result implies that either paternal leakage is rare or that paternal mtDNA is transmitted to the progeny below our detection limit (0.3%).

**Fig. 2:**
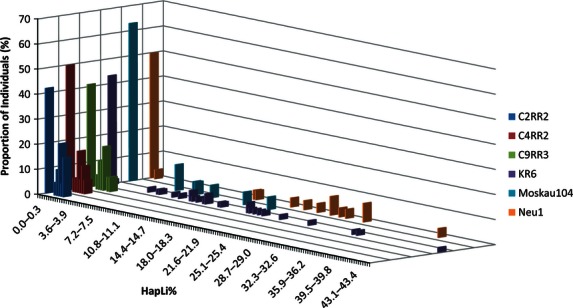
Distribution of heteroplasmy levels among offspring of three experimental crosses (C2RR2, C4RR2 and C9RR2) and three wild-caught females (KR6, MOSKAU104 and NEU1). The graph shows the percentage of individuals (*Y*-axis) carrying a certain level (*X*-axis) of a second mtDNA haplotype.

To study the transmission of the haplotypes from heteroplasmic mothers, we determined the distribution of heteroplasmy levels in siblings (males and females) of three individuals (KR6, MOSKAU104 and NEU1). All flies analysed (a total of 71) were F1 progeny of wild-caught females, and therefore, they reflect the distribution of heteroplasmy found in nature. However, to estimate the drift of mtDNA haplotypes, we need to be able to empirically determine the level of heteroplasmy in the mother. As none of the three wild-caught females were available, we also analysed the progeny of three heteroplasmic females (C2RR2, C4RR2 and C9RR3) from isofemale lines established in 2004 (EVO population, see Materials and methods for details). Figure 2 (and Table 1 for a summary of the data) shows the distribution of heteroplasmy in all six families. Interestingly, considerable heterogeneity in haplotype frequency was detected among offspring, and in all cases more than 40% of the offspring have no detectable heteroplasmy (Fig. 2). The distribution of heteroplasmy among progeny is not significantly different between sexes within and across lines (*P* > 0.05, Median one-way analysis). In addition, we found no significant difference in the distribution of heteroplasmy among progeny of the three wild-caught females and the three laboratory-adapted lines (*P* > 0.05, Median One-way Analysis). In fact, there is no significant difference between the six different lines within and across sexes. Note that due to the moderate sample sizes, we cannot rule out that minor differences exist between the samples. The level of heteroplasmy in the offspring of the laboratory-adapted lines is not significantly different from that of the corresponding mother (*P* > 0.05, Median one-way analysis). As this is the expected pattern for neutral drift, our results suggest no strong selection against one of the two haplotypes in heteroplasmic individuals. The heterogeneity in haplotype frequencies among offspring indicates considerable drift during mtDNA transmission.

**Table 1:** Descriptive statistics for the distribution of heteroplasmy in the progeny of heteroplasmic females

	Frequent Allele/COI hap*	HapLi†	%HapLi in F0	Sex‡	*N*	Median	25%Q1	75%Q3	Range
Experimental crosses									
C2RR2	T/2	A	0.4	M	19	1.6	0.0	2.0	0.0–2.5
C4RR2	T/2	A	0.6	M	18	0.6	0.0	1.7	0.0–2.6
C4RR9	T/2	A	0.5	M	17	1.6	0.0	2.2	0.0–3.0
Natural populations									
KR4	T/2	A	NA	M	27	8.7	0.0	15.3	0.0–33.8
	T/2	A	NA	F	19	0.5	0.0	19.1	0.0–45.1
MOSKAU4	A/1	T	NA	M	12	0.0	0.0	6.4	0.0–15.8
	A/1	T	NA	F	7	0.1	0.0	9.1	0.0–18.9
NEU1	A/1	T	NA	M	14	16.8	0.0	24.5	0.0–29.2
	A/1	T	NA	F	12	0.0	0.0	18.6	0.0–39.2

M, male; F, female; *N*, sample size; NA, nonapplicable.

Median, 1st and 3rd quartiles and range of the distribution of %HapLi among progeny.

* Maternal (as determined by sequencing of the COI fragment).

† Rare allele as determined by RT-qPCR.

‡ Sex of the progeny analysed.

**Fig. 3:**
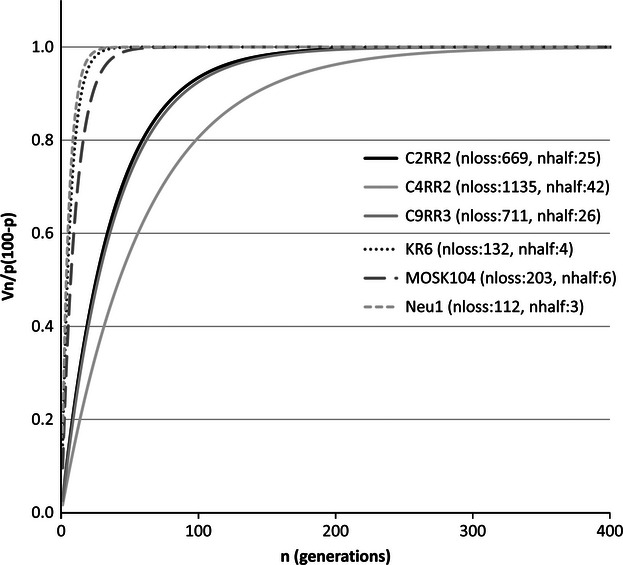
Number of generations (*nloss*) required for complete segregation of mtDNA haplotypes. Complete loss of heteroplasmy is achieved when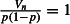. nhalf is the number of generations necessary to decrease by half the difference between the initial and the final variance.

As our data suggest that the transmission of heteroplasmy is a random process, we can estimate the time, in number of generations (*n*), required for all the descendants of a heteroplasmic female to return to the homoplasmic state (*nloss*) using a modification of Wright's genetic drift model (Wright 1968). We also calculated *nhalf*, which is the number of generations necessary to decrease by half the difference between the initial and the final variance. The results are shown in Fig. 3. The time (in generations) required for complete segregation of mtDNA haplotypes in all the descendants of a heteroplasmic female (*nloss*) is variable between lines because they have different variances. In all cases, at least 100 generations are necessary to return to the homoplasmic state. This means that heteroplasmy can be maintained for a long time before it is completely lost.

The observed frequency of heteroplasmy in natural populations is the outcome of a balance between paternal leakage, creating new heteroplasmic individuals, and heteroplasmy loss, restoring homoplasmic states. Hedrick's forward and backward mutation equation can be used to calculate the paternal leakage. Based on our estimate of the equilibrium frequency of heteroplasmic individuals (*q*_*e*_ = 0.14) and the minimum of loss of heteroplasmy among the six families tested (*v* = 0.4), we inferred a rate of paternal leakage in natural populations of *D. melanogaster* of 6%. We note, however, that the precision of our paternal leakage estimate depends on the accuracy of *q*_*e*_ and *v*.

## Discussion

In this study, we took advantage of the high haplotypic diversity in Europe and quantified the frequency of heteroplasmy in natural *Drosophila melanogaster* populations. The number of heteroplasmic individuals (14%) was strikingly high given that it has so far remained unnoticed. This is most likely explained by very low levels of the second haplotype, which complicated the detection in previous studies. Furthermore, our estimate is extremely conservative because many more combinations of haplotypes not discernible by the RT-qPCR assay are possible, as evidenced by our data (we found heteroplasmy involving haplotypes indistinguishable by our assay, in four individuals: KR15, ROME3, GOT11 and TU19). Our results are particularly noticeable in the light of recent findings indicating that in *D. melanogaster*, mtDNA is actively eliminated during spermatogenesis (DeLuca & O'Farrell 2012). One possible explanation for this discrepancy is that the mechanisms responsible for elimination of sperm mtDNA are not as tightly regulated in nature as they might be in the laboratory, because of environmental stress or differences in the genetic background, but this remains to be tested. We studied the transmission of heteroplasmy in the offspring of three wild-caught females as well as in the offspring of three lines maintained in the laboratory for several years. The distribution of heteroplasmy levels among the offspring does not differ significantly. However, our data are quite scattered (Fig. 2), and although our test statistics were all far from significant, it is possible that a substantial increase in sample size may detect differences in the transmission of mtDNA haplotypes between genotypes. In addition, we found no evidence for strong selection against one of the two haplotypes in heteroplasmic individuals neither in the transmission study nor in the heteroplasmy frequency screen in natural populations. Given the high loss of (detectable) heteroplasmy among offspring and the observed frequency of heteroplasmic individuals in natural populations, we estimated that the rate of paternal leakage must be at least 6%. This estimate could be inflated by an overestimation of the rate of heteroplasmy loss inferred from the transmission study due to the detection limit of our RT-qPCR. However, this would also cause an underestimation of the frequency of heteroplasmy in natural populations (i.e. *q*_*e*_), which in turn would decrease the estimate of paternal leakage.

Our estimate of paternal leakage is much lower than that detected in experimental crosses between *Drosophila simulans* strains carrying divergent haplotypes: Sherengul *et al*. (2006) found evidence for paternal leakage in 32–48% of the backcrosses. The authors used a backcross and extracted DNA from pools of individuals, which may have facilitated the detection of paternal mtDNA. The recent study of Wolff *et al*. (2013), also in *D. simulans*, is probably more comparable to ours because the presence of paternal mtDNA was tested in F1 progeny. In contrast to Sherengul *et al*. (2006), they found that paternal leakage occurred only in 3% of the crosses, which is much closer to our estimate.

Despite the high levels of heteroplasmy exhibited by flies collected in nature, we were unable to reproduce paternal leakage in experimental crosses. As frequency of paternal leakage may vary between lines, we decided to include as many mtDNA–ncDNA combinations as possible to avoid biased results. Screening many individual progeny from many mtDNA–ncDNA combinations would have shed light on heterogeneity of paternal leakage; however, this is something that was outside the scope of this manuscript. As the rates of paternal leakage for particular mtDNA–ncDNA combinations are unknown, we reasoned that we would have more power by including as many different mtDNA–ncDNA combinations as possible. Hence, we used a population with unambiguous evidence for paternal leakage and screened several genotypes with a moderate number of progeny per genotype. However, as our estimate of paternal leakage is based on a population average, we pooled the offspring from different experimental crosses. With a paternal mtDNA leakage rate of 6% and the number of crosses and offspring analysed (29 and 116 respectively, ignoring crosses with the mother being heteroplasmic), we expect enough power to detect paternal leakage (*P* < 0.01, binomial distribution). While this could mean that we overestimated the rate of paternal leakage, we do not think that this is very likely (see Results section). Alternative explanations are as follows: (i) paternal mtDNA is transmitted to the progeny below our detection limit, (ii) laboratory conditions cannot reproduce the natural environment of the flies, and (iii) the rate of paternal leakage is genotype dependent and we used too few genotypes to detect this.

In the last 10 years, evidence for animal mtDNA recombination has accumulated (Ladoukakis & Zouros 2001; Kraytsberg *et al*. 2004; Piganeau *et al*. 2004; Gantenbein *et al*. 2005; Tsaousis *et al*. 2005; Ciborowski *et al*. 2007; Ujvari *et al*. 2007). Despite the high frequency of heteroplasmy, we were not able to find evidence for recombination among the different mtDNA haplotypes. This result is surprising because mitochondria have the necessary machinery to recombine (Thyagarajan *et al*. 1996; Lakshmipathy & Campbell 1999a,b). This could be due to biological reasons, such as selection against recombinant molecules, or because maternal and paternal mtDNA are kept in different nucleoids (nucleoproteins–mtDNA complexes) that do not mix or exchange genes (but see D'Aurelio *et al*. 2004). We note, however, that we cannot entirely rule out that we missed recombinant molecules due to technical limitations of our study. Particularly, the sequencing of PCR products may fail to detect low-frequency recombinant molecules. Single-molecule sequencing techniques enabling long sequence reads will be the method of choice to identify low-frequency recombinant mtDNA molecules.

*Wolbachia* is a maternally inherited endosymbiont. A recent phylogenetic study of the mitochondrial and *Wolbachi*a genome identified a high congruence between the phylogeny of both molecules, suggesting a strict vertical transmission of both molecules (Richardson *et al*. 2012). Assuming a stochastic fixation of either haplotype in heteroplasmic individuals, this is expected to result in phylogenetic incongruence. We propose two alternative explanations to reconcile the high heteroplasmy observed in our study with the phylogenetic congruence observed by Richardson *et al*.(2012). First, the fixation of mtDNA haplotypes may not be random, but paternal haplotypes are preferentially lost. Second, Richardson *et al*.(2012) predominantly included samples from geographic regions that harbour mainly similar mtDNA haplotypes. Hence, paternal leakage may therefore affect only similar haplotypes with no detectable phylogenetic incongruence. We anticipate that phylogenetic analysis of mtDNA and *Wolbachia* from *D. melanogaster* collected in the Mediterranean will shed further light on this controversy.

## Conclusions

It is difficult to evaluate the evolutionary consequences of the heteroplasmy of mtDNA in *Drosophila melanogaster*. While the lack of detectable recombination and selection for a particular mtDNA haplotype would suggest that phylogeographic and demography studies should not be highly influenced by paternal leakage in this species, we caution that it has been previously noted that even low levels of paternal mtDNA may be sufficient to affect the levels of mtDNA variability and population differentiation (Takahata & Maruyama 1981; Kondo *et al*. 1990). Furthermore, it may contribute to signatures over larger evolutionary timescales (Ladoukakis & Zouros 2001; Tsaousis *et al*. 2005). In fact, although the average level of heteroplasmy is very low, we did find flies with more than 40% of the minor haplotype (Fig. 2), and therefore, it is possible that sometimes the paternal mtDNA replaces the maternal one. Furthermore, recombination might happen too rarely to be detected but sufficiently frequent to prevent mutation accumulation (Charlesworth *et al*. 1993; Barr *et al*. 2005).

## Data accessibility

DNA sequences: GenBank accessions EF153514–EF153618, FJ158857–FJ158959, JX458136–JX458325, JX467036–JX467121 and JX475468–JX475894 and Table S1 (Supporting information).

Concatenated haplotype and COI alignment files uploaded as online Supporting information (Alignment Files S1 and S2, respectively).

Input data file for analysis of the distribution of heteroplasmy among progeny of a heteroplasmic female uploaded as online Supporting information Table S6.

## Supporting information

**Fig. S1** COI sequence chromatogram from KR6.

**Fig. S2** Haplotype network of seven concatenated mtDNA fragments sequenced from 85 individuals.

**Table S1** List of samples sequenced either for COI alone or all seven mtDNA fragments.

**Table S2** List of primers used for PCR and sequencing of mtDNA fragments.

**Table S3** List of primers and probe used in the quantification of heteroplasmy by allele specific RT-qPCR.

**Table S4** Results of the heteroplasmy screen in natural populations using allele specific RT-qPCR.

**Table S5** Experimental crosses to test for rate of paternal leakage.

**Table S6** Distribution of heteroplasmy among progeny of heteroplasmic females.

**Table S7** Diversity estimates for a 554 bp fragment of the mt: COI gene in natural populations of *Drosophila melanogaster*.

**Alignment File S1** Concatenated haplotype file.

**Alignment File S2** COI alignment file.
