# Supplementary material for: Extensive paternal mtDNA leakage in natural populations of Drosophila melanogaster
Source: Mol Ecol. 2013 Mar 4;22(8):2106–17. doi: 10.1111/mec.12256 (PMC3659417; doi:10.1111/mec.12256)
Supplement: Supplementary file 1 [file mec0022-2106-SD1.pdf]

Traces from Unassembled Samples

Print | Previous Feature | Next Feature | Colors | Bases<>Transl. | Next Frame | Mask Matches | Help

TTTTTTATr GTTATACC TATTATAATT GGTGGATTT GGAAATT GATTAGTGCC TTTAAT ATTAGGT GCTCC TG ATATAGCATTCCC ACG AATAAAT AAT ATAAGATTTT GACT wCTACC

Kr6_48F.abd

40 50 60 70 80 90 100 110 120 130 14

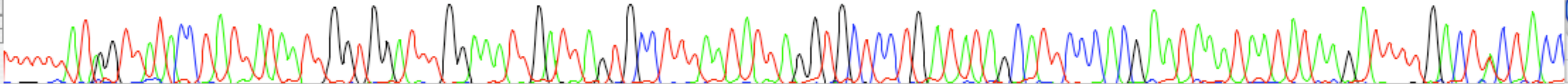

Kr6_48F.abd

Base 1 of 620

Quality: 15
