# Supplementary figures and images for: Extensive paternal mtDNA leakage in natural populations of Drosophila melanogaster
Source: Mol Ecol. 2013 Mar 4;22(8):2106–17. doi: 10.1111/mec.12256 (PMC3659417; doi:10.1111/mec.12256)

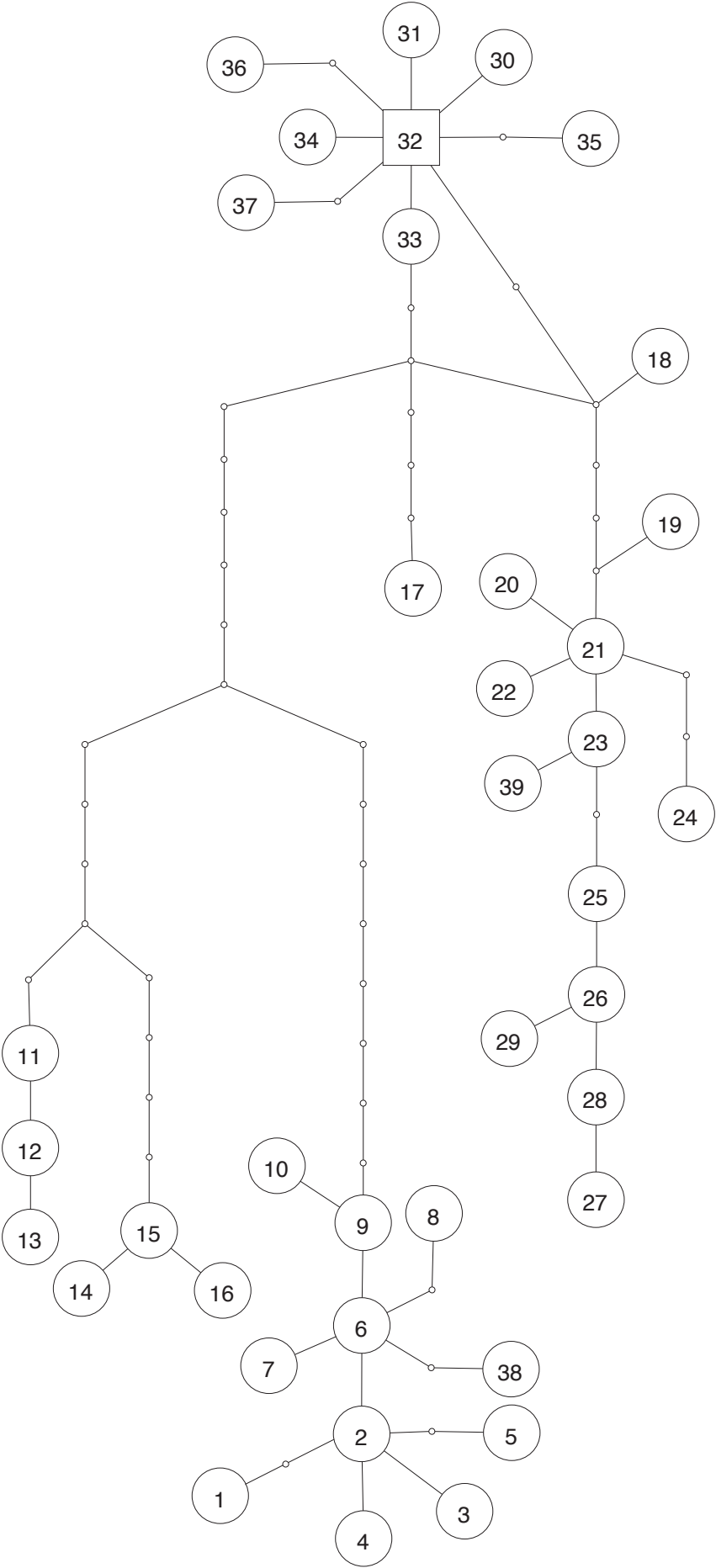

Supplement: Supplementary file 2 [file mec0022-2106-SD2.pdf]
